# IgG4-Related Fibrotic Diseases from an Immunological Perspective: Regulators out of Control?

**DOI:** 10.1155/2012/789164

**Published:** 2012-06-04

**Authors:** Laura C. Lighaam, Rob C. Aalberse, Theo Rispens

**Affiliations:** ^1^Landsteiner Laboratory, Academic Medical Centre, University of Amsterdam, 1066 CX Amestrdam, The Netherlands; ^2^Sanquin Blood Supply Foundation, Plesmanlaan 125, 1066 CX Amsterdam, The Netherlands

## Abstract

Patients with autoimmune pancreatitis have a striking polyclonal elevation of total IgG4 in serum. This observation has been confirmed and extended to other fibrotic conditions (that are therefore called IgG4-related disease) but as yet remains unexplained. The affected tissue contains many IgG4-producing plasma cells embedded in a fibrotic matrix originating from activated mesenchymal (stellate) cells. We propose that the process results from an unusual interaction between two regulatory systems: the regulatory arm of the immune system (including Bregs) and the tissue repair regulatory components orchestrated by the activated stellate cell. This interaction results in ongoing mutual activation, generating TGFbeta, IL10, and vitamin D. This environment suppresses most immune reactions but stimulates the development of IgG4-producing plasma cells.

## 1. IgG4 Production in IRD

IgG4-related disease (IRD, see Box 1) is a group of diseases with disparate symptoms, but sharing a common pathophysiology, which has only recently been recognized as a new disease entity [1]. IRD is characterized by massive infiltration of the affected organ by IgG4-positive plasma cells. This infiltration coincides with a disruption of the organization of the tissue and thus of tissue function. The extent of the plasmacytic tissue reaction in IRD is such that the first impression is often that of a tumor. While the prototypic site of IgG4 production in IRD is the pancreas, many other sites in the body can be involved, for example, the salivary and tear glands, reminding of Sjögren's syndrome. However, in IRD, the ducts usually remain largely intact, and secretion by the glands is less severely affected [2]. It is not at all unusual to find several organs to be involved simultaneously (for details, see Box 1).

A 5–50-time elevation of total IgG4 levels is found in patients with IRD. This results in a markedly increased IgG4/IgG ratio, both for serum immunoglobulin levels and for plasma cells in the affected tissue. It is not clear if the increased levels of IgG4 contribute to the pathology of IRD. So far, convincing support for the hypothesis that (auto-) antibody activity of IgG4 is driving the pathology is lacking. Several candidate autoantibodies have been suggested in IRD, such as antibodies directed against pancreatic trypsin inhibitor, lactoferrin, and carbonic anhydrase, mainly in patients with pancreatic involvement [3]. These antibodies were mostly not of the IgG4 subclass. Since they are present in only a small part of the patients, their role in the pathophysiology of the disease is probably limited. In the absence of an obvious (auto) antigen driving the reaction, it is unclear how these responses are triggered, and, therefore, how IRD may develop.

Toll-like receptor and Nod-like receptor stimulation have also been implied in IRD, since PBMCs of IRD patients produce IL-10 and high levels of IgG4 in response to stimulation of these receptors in a BAFF-dependent manner [4, 5].

Recently, some IRD patients have been treated with Rituximab, a monoclonal antibody drug that targets CD20 [6]. Patients treated with Rituximab show a fast decline in serum IgG4 levels, while the decrease of other subclasses is less pronounced [7]. This is not due to a direct effect on the IgG4-producing plasma cells, because CD20 is present on B cells from the pre-B cell stage, but is lost upon differentiation into plasma cells. Therefore, the rapid decline of IgG4 levels upon B-cell depletion strongly suggests that the lifespan of the IgG4-secreting plasma cells is short, that is, less than a week. The large number of IgG4-secreting plasma cells before treatment must be caused by the continuous differentiation of IgG4-switched B cells into plasma cells.

Here, we will discuss two features related to IgG4 that may be involved in the preferential recruitment and retention of IgG4-switched B cells into the affected tissue in IRD. First, as explained below, IgG4 has been linked to “tolerogenic” immune responses. Second, there are indications of unusual Fab glycosylation in (part of) IgG4. Our hypothesis is that the B-cell receptors (BCRs) of some B cells are Fab glycosylated with an oligomannose glycan, which is recognized by an endogenous lectin found on the tissue-resident myofibroblast (stellate cell). This interaction may result in an ongoing mutual stimulation of two regulatory systems: the blood-derived immune regulators, including IgG4-committed B cells, and the tissue-resident damage-controlling stellate cell, resulting in the pathology observed in IRD.

## 2. IgG4: An Antibody Linked to Tolerogenic Conditions

IgG4 is a peculiar subclass of human immunoglobulins. It represents about 5% of total IgG in serum of healthy adults (0.5 g/L, normal range: 0.05–1.4 g/L). However, IgG4 antibody can represent up to 80% of total IgG antibody after chronic exposure to antigen [8, 9]. Since IgG4 antibodies do not activate complement and bind to Fc receptors with lower affinity [10], they do not activate the effector functions of the immune system in the same way the other subclasses do [11, 12]. Furthermore, IgG4 antibodies are able to exchange half molecules *in vivo* [12, 13]. This process results in the generation of asymmetric antibodies with two different Fab arms. Since these antibodies can, in general, only bind to antigen with one Fab arm, IgG4 is not able to cross-link antigens and thus to form large immune complexes. IgG4 has even been shown to interfere with the complement-activating and immune-precipitating activities of human IgG1 antibodies [14].

All in all, the immunochemical properties of IgG4 antibodies point towards a dampening role in the effector phase of the immune response. This fits well with the requirements for IgG4 production. IgG4 responses require frequent and/or high antigen exposure and are observed in situations associated with tolerance induction, such as during immunotherapy. IgG4 responses are also often associated with IgE-mediated allergy, but IgG4 responses are distinct from IgE responses. Although both IgG4 and IgE need the Th2 cytokines IL-4 and/or IL-13 [15], production of IgE antibodies often occurs well before IgG4 antibodies appear (e.g., in novice beekeepers [8]). It is also common to find IgG4 antibodies in the absence of IgE antibodies, a process called the modified Th2 response [16]. One important regulatory component in the modified Th2 response is IL-10. Under the influence of this cytokine, the switch to IgE is inhibited, while switch to IgG4 is promoted [17].

In the case of prolonged and/or high-dose antigenic stimulation, immune regulatory circuits play an important role. They counteract the effects of antigenic stimulation and dampen the immune response, resulting in, amongst others, the decrease of T_effector_ responses and of the production of human IgG1 antibodies. It is only then that the IgG4 response develops to its full extent. One of these regulatory signals is the above-mentioned cytokine IL-10. This explains why upon chronic exposure to antigen, IgG4 levels increase. It is likely that IL-10 needed for the development of an IgG4 immune response is in part produced by Tregs present in the lesions of IRD patients as demonstrated by *in situ* hybridization [18], as well as increased levels of circulating Tregs [19]. Besides Tregs, another likely source for IL-10 is regulatory B cells (for a review on regulatory B cells, see [20]), some of which may later develop into IgG4-producing cells [21]. 

## 3. IgG4 Fab Glycosylation

There are indications that IgG4 may sometimes be unusually glycosylated in the Fab region: two sets of information point to a link between IgG4 and Fab glycosylation of the oligomannose type. First, a subject that has been studied for many years by Margni and coworkers is the association between oligomannose-type Fab glycosylation and nonprecipitating antibodies. They fractionated antigen-specific polyclonal antibodies based on their glycosylation pattern by ConA lectin chromatography, which preferentially binds oligomannose glycans. The bound fraction was unable to form an immune precipitate with antigen. The lack of immune precipitation was found to be due to asymmetric Fab glycosylation, that is, glycosylation of only one of the two antigen-binding domains. A possible mechanism explaining the formation of asymmetrically glycosylated antibodies is the aforementioned Fab arm exchange of IgG4. Fab arm exchange between glycosylated and nonglycosylated IgG4 would result in a nonprecipitating asymmetrically glycosylated antibody. Conditions that lead to enhanced production of asymmetrically glycosylated antibody (such as pregnancy) are similar to the tolerizing conditions that promote IgG4 production. These data suggest that IgG4 might be preferentially Fabglycosylated with oligomannose glycans.

The other set of information comes from a study on IgG4 antibody responses in infancy to a panel of food allergens [22]. In this study, a strong reactivity to a protein in banana was found, which was then characterized and found to be a lectin with a preference for oligomannose glycans: BanLec1 [23, 24]. IgG, including IgG4, is a glycoprotein. The obvious question was whether BanLec1 bound to a glycan on IgG4, or whether IgG4 reacted as a genuine antibody with a protein that happened to be a lectin. At that time, IgG glycosylation was generally assumed to be restricted to the Fc part and was of the complex glycan type. When we found that the binding of BanLec1 to IgG4 was restricted to the Fab part, we considered this to be a strong argument in favor of IgG4 binding as an antibody, rather than as a glycoprotein. However, these recent data make us uncertain about the interpretation of our earlier results, and research is currently carried out to further explore the glycosylation of IgG4. 

## 4. Lectin-Driven B-Cell Activation

As already mentioned, because of the highly elevated levels of IgG4 in serum of IRD patients (typically more than 5 g/L), we consider it unlikely that the signal for activation of the IgG4^+^ B cell is a regular antigen. The above-mentioned indications of unusual glycosylation of the Fab of (part of) IgG4 suggest that, instead, an endogenous lectin may function as an alternative trigger of the BCR. The B cell would be activated by the lectin upon cross-linking of the BCR via its Fab glycan. In a way, the lectin would act as an endogenous superantigen, resulting in recruitment of IgG4-switched B cells in particular.

Support for an “oligomannose Fab glycan + endogenous lectin” scenario for IRD comes from the work of Stevenson and coworkers on the activation of B cells in follicular lymphoma (FL). They found that the majority of FL cases involve a mutation resulting in the incorporation of a glycan acceptor site in the variable region of the Ig [25]. They showed that the binding of mannose-binding lectin to the FL cells triggers BCR-mediated signaling. These cells do not need to recognize antigen anymore to proliferate, giving them a major growth advantage. Furthermore, they found that the glycan attached to the Fab arm in these follicular lymphomas is terminated in oligomannose, an uncommon structure for human glycoproteins. Interestingly, cases of IRD are sometimes mistaken for FL due to similarities between these two diseases. In both IRD and FL, B-lymphocytes invade tissues and extensively proliferate there. However, in IRD the cells differentiate into plasma cells, whereas in FL they typically do not, making high serum levels of IgG4 a diagnostic marker to distinguish IRD from lymphoma. Furthermore, the IgG4 cells in IRD are of polyclonal origin, in contrast to the monoclonal B cells in FL.

## 5. A Model for IgG4 Plasma Cell Development in IRD

**Figure 1:**
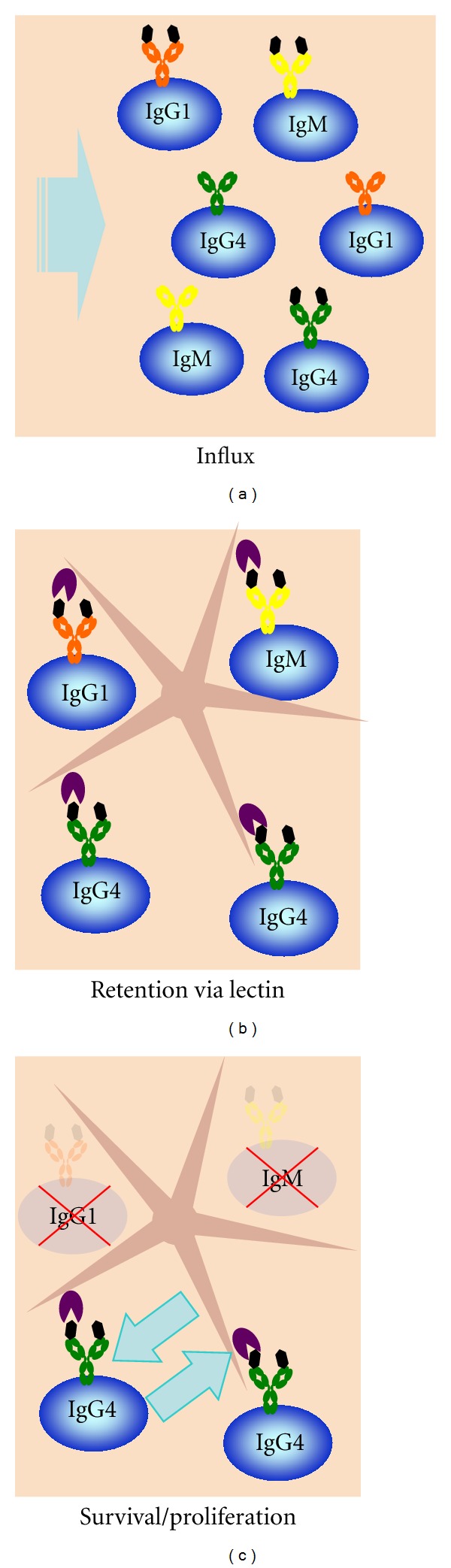
Proposed model of B-cell infiltration into affected tissue. (a) B cells from the circulation enter the inflamed tissue. (b) Differential glycosylation of IgG4-switched B cells allows retention and activation of this cell type via an as-yet-unidentified lectin on the stellate cell. (c) In this model, the local environment of the affected tissue will further promote survival/proliferation of IgG4-switched B cells due to a tolerogenic environment that may in part be created via signals from the IgG4 B cells themselves.

Based on the requirements for IgG4 production and the link between IgG4 and Fab oligomannose glycans, we propose a model in which B cells in circulation are entering into the inflamed tissue, where IgG4 cells are preferentially retained and differentiate in the tolerogenic environment of the lesion (Figure 1). The initial sequence is presumably a traumatic or infectious event that triggers a repair response. In case of pancreatitis, the local repair reaction in the tissue is orchestrated by the pancreatic stellate cell (see Box 2), which results in a storiform fibrotic reaction, one of the hallmarks of IRD. This results in the attraction and entrapment of circulating regulatory cells, including Tregs and Bregs, together creating a “tolerogenic” environment characterized amongst others by cytokines like IL-10 and IL-21, as well as vitamins A and/or D released from the activated stellate cells. In this environment, interaction of infiltrated IgG4-switched B cells with an as-yet-unidentified-lectin in combination with the tolerogenic conditions leads to differentiation of these B cells to plasma cells (but also see Box 3). On the other hand, proliferation/differentiation of other B cells is disfavored. Support in favor of this scheme comes from the IgG4 plasmacytosis seen in myofibroblastic tumors [26, 27]. This repair process should be self-limiting, but somehow this feedback is not working, and a feed-forward reaction is initiated. To stop this feed-forward loop, not only the B cells but also the stellate cells may need to receive signals to terminate their “repair mode” that sustains the local tolerogenic conditions. This could explain why anti-CD20 B cell depletion therapy with Rituximab needs to be perpetuated: it targets the B cells but leaves the pancreatic stellate cell unaffected. How can we regulate the regulators?

## 6. Perspectives

Some of the many questions that need to be answered are the following: (1) upon stimulation, is the stellate cell capable to initiate or increase the production of the hypothetical oligomannose-specific lectin, and of chemokine receptor ligands that attract Tfh and class-switched B cells? Possible involvement of BCR stimulation via oligomannose could be studied *in vitro,* for example, using BanLec-1, something that is currently being pursued in our lab; (2) what is the phenotype of the T cells (e.g., Tfh or Treg) and B cells (e.g., IL-10 producing and/or IgG4 switched; glycosylation status of BCR) within the affected organ?; (3) what is the relation between the lymphocytes in the tissue and those in the blood, particularly in relation to their chemokine receptors and (for the B cells) their Fab glycosylation profile?

## Figures and Tables

**Box 1:**
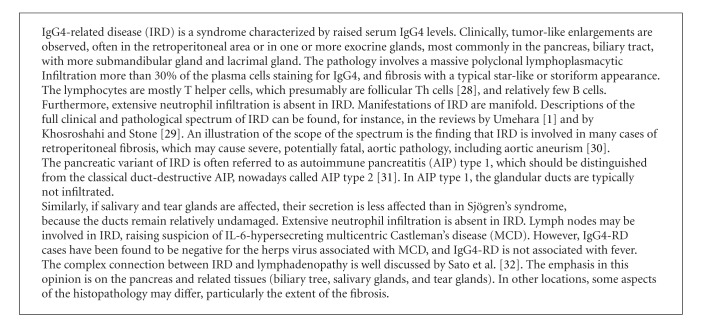
IgG4-related disease (IRD).

**Box 2:**
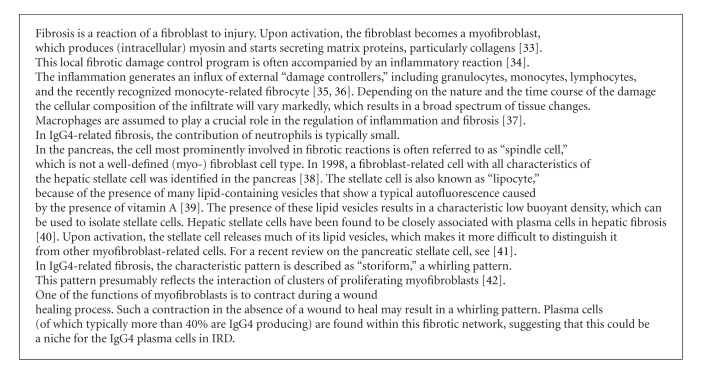
The role of the myofibroblast-type stellate cell in fibrosis, tissue repair and plasma cell differentiation.

**Box 3:**
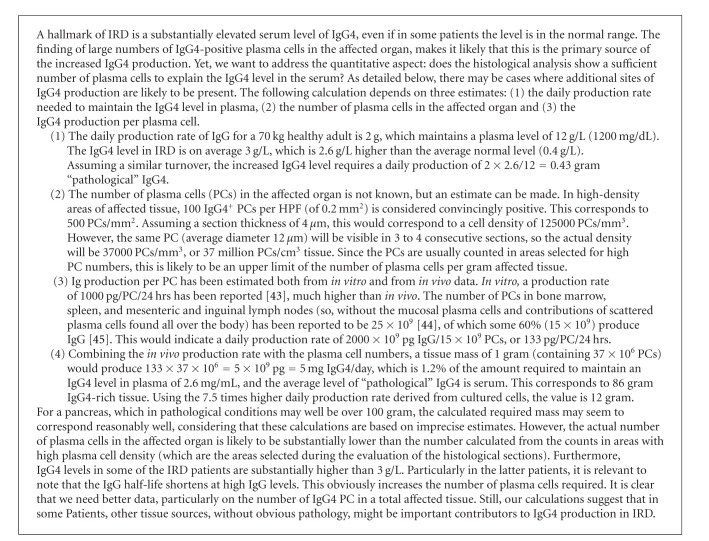
A quantitative conundrum: the number of tissue-residing plasma cells is insufficient to explain the strongly elevated IgG4 level in plasma.
